# Proton pump inhibitor use does not increase dementia and Alzheimer’s disease risk: An updated meta-analysis of published studies involving 642305 patients

**DOI:** 10.1371/journal.pone.0219213

**Published:** 2019-07-02

**Authors:** Ya Qi Song, Yong Li, Su Li Zhang, Jie Gao, Shun Yi Feng

**Affiliations:** Emergency Department, Cangzhou Central Hospital, Yunhe Qu, Cangzhou, China; Istituto Di Ricerche Farmacologiche Mario Negri, ITALY

## Abstract

Recent studies have indicated an increased risk of dementia and Alzheimer’s disease (AD) among people who consume proton pump inhibitors (PPIs), but the results of those studies are inconsistent. This meta-analysis aimed to explore the correction risk of dementia and AD among PPI users. The literature search for relevant studies was conducted in PubMed, Web of Science, EMBase and ScienceDirect. Pooled hazard ratio (HR) and 95% confidence intervals (CIs) were used to assess the relationship between the PPIs and risk of dementia and AD. Ten independent studies that involved 642305 participants were included in this meta-analysis. PPI users were unassociated with dementia (HR = 1.04, 95% CI 0.92–1.15; *I*^2^ = 95.6%, *p* < 0.001) and AD (HR = 0.96, 95% CI 0.83–1.09; *I*^2^ = 80.7%, *p* <0 .001). No evidence of publication bias was detected by Begg’s and Egger’s test. Sensitivity analyses showed no important differences in the estimates of effects. The current evidence indicates that PPI use does not increase dementia and AD risk. The remarkable heterogeneity among the studies warrants a further review of our findings.

## Introduction

The introduction of proton pump inhibitors (PPIs) into clinical practice has revolutionised the management of acid-related diseases. They are extensively used to treat acid-related disorders, such as dyspepsia, gastrooesophageal reflux disease, Zollinger–Ellison syndrome, Barrett’s oesophagus and *Helicobacter pylori* infection of the upper gastrointestinal tract. Since their release in the late 1980s, PPIs have become some of the most widely prescribed agents in outpatient and inpatient settings worldwide [1, 2].

Prescribing these agents for long-term use has become a common clinical practice [3, 4]. Moreover, PPIs are currently over-the-counter medications in many countries; therefore, their use is often not monitored by a healthcare specialist. Notable, side effects of long-term use of PPIs are gaining increasing attention, especially cognitive impairment [5–9]. Wijarnpreecha et al.[10] performed pooling analyses regarding the association of dementia and PPI users, and indicated an increased risk of dementia and Alzheimer’s disease (AD) among PPI users. Nevertheless, several subsequent studies were not included in these meta-analyses, and these studies had shown inconsistent conclusions[6, 11–16]. Hence, the association of PPIs and dementia and AD remains unknown before re-evaluating the susceptibility effects. Herein, we summarise the data from more studies to perform a meta-analysis for re-evaluating the association of PPIs and dementia and AD.

## Materials and methods

### Study design and registration

This systematic review was performed according to the guidelines of Preferred Reporting Items for Systematic Reviews and Meta-analyses [17]. Ethical approval and informed patient consent were not required, given this study was a literature review and had no direct patient contact or influence on patient care. The protocol was published by Centre for Reviews and Dissemination PROSPERO (Registration No. CRD42018117727).

### Search strategy

We searched PubMed, Web of Science, EMBase, ScienceDirect and studies published up to November 1, 2018. The following key words were used in the search queries: ‘proton pump inhibitors’ and ‘dementia.’ Moreover, reference lists of relevant published literature were manually checked to identify additional eligible meta-analyses.

### Inclusion and exclusion criteria

Publications were considered eligible for this meta-analysis when they fit all of the following criteria: studies assessed the association between PPIs and risk of dementia; studies that had access to full texts; studies on clinical trials in humans and studies reported sufficient data necessary to calculate the hazard ratio (HR) and its 95% confidence interval (CI). The following studies were excluded: duplicates or multiple publications of the same study; abstracts; case reports; commentaries; animal studies and conference papers.

### Data collection and quality assessment

Two independent reviewers screened the data from the included studies using a predefined checklist for each study. The following information of the meta-analyses was extracted: first author’s surname, publication year, study design, sample size, male percentage, age and risk estimate (95% CI). The Newcastle–Ottawa Quality Assessment Scale (NOS) [18, 19] was used to assess the quality of the studies included in the meta-analysis and performed by two reviewers with a third reviewer consulted in case of discrepancy. The scale ranged from 0 to 9 points, namely, 4 for selection of participants, 2 for comparability among groups and 3 for assessment of outcome, with higher scores indicating higher study quality. NOS scores ranged from 0 to 9, and a score of 7 or greater indicated high quality [18].

### Statistical analysis

All analyses were conducted by using Stata 14.0 (StataCorp, College Station, TX). The relative effect results for the consistency model were reported as an HR with a corresponding 95% CI. The assessment for statistical heterogeneity was calculated using the chi^2^ and *I*^2^ statistics. If no heterogeneity (*p*> 0.05, *I*^2^<50%) existed, a fixed-effect model was used. Otherwise, a random-effect model was used. Metaregression, subgroup analyses and sensitivity analyses were conducted to determine the possible heterogeneity causes and to identify the influences of various exclusion criteria on the overall risk estimate. We investigated the possibility of publication bias through funnel plots and the statistical evaluation of Egger’s and Egger’s regression [20]. Two-tailed *p* values < 0.05 were considered statistically significant.

## Results

### Search strategy

**Fig 1:**
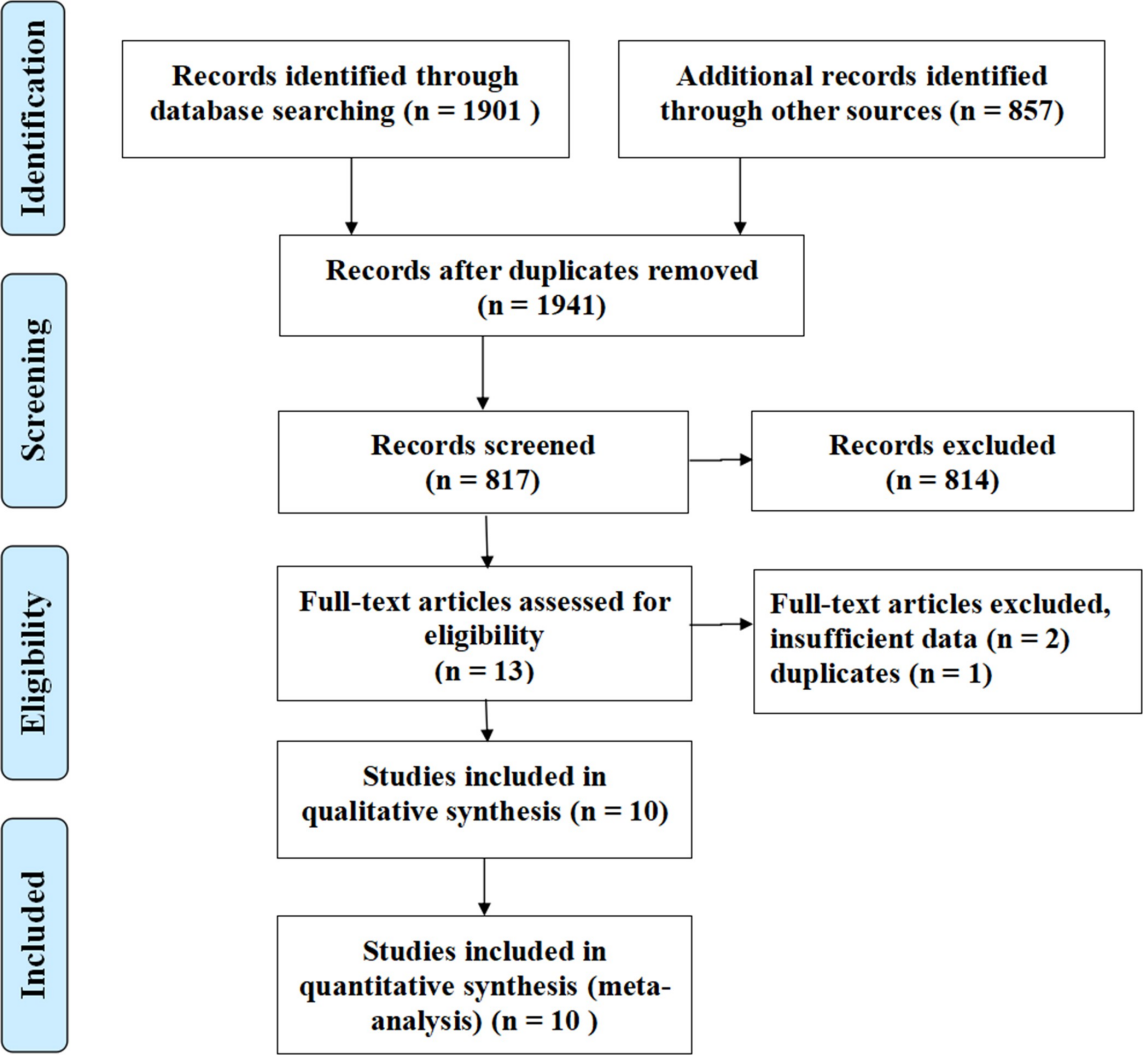
Flowchart of the selection process for studies.

The flow diagram of identification and selection of meta-analysis and reference bibliography is shown in Fig 1. Initially, 2758 articles were identified, and 1941 duplicates were excluded. Subsequently, 814 irrelevant records were removed through primary screening of titles and abstracts, and 13 full-text studies were assessed for eligibility. Finally, we included 10 clinical trials [5, 6, 11–16, 21, 22] in the meta-analysis.

### Study characteristics

The characteristics of each study included into this meta-analysis are presented in Table 1. Sample size of each eligible study incorporated into our study varied from 3076 to 353576 and in total of 642305 participants. Three studies were conducted in Germany, and one study came from France, Georgia, Korea, USA, the UK, Taiwan and Finland. The scores of the eligible studies from the NOS ranged from 8 to 9, with a mean of 8.6, indicating that the included studies were of high quality.

**Table 1:** Characteristics of included studies.

Author (Year)	Region	Design	N	Male (%)	Age (Year)	Risk estimate (95% CI)	NOS score
**Barreto et al. 2013 [21]**	France	Cross-sectional study	6275	26.3	86	Dementia:0.666 (95% CI 0.588–0.755)	8
**Booker et al. 2016 [22]**	Germany	Case-control study	23912	39.0	80.4	Dementia: 0.93 (95% CI 0.90–0.97)	8
**Goldstein et al. 2018 [11]**	Georgia	Observational, longitudinal study	10486	38.5	72.9	Dementia:0.82 (95% CI 0.69–0.98) AD: 0.82 (95% CI 0.69–0.98)	8
**Gomm et al. 2016 [5]**	Germany	Prospective cohort study	73679	26.3	83.0	Dementia:1.44 (95% CI 1.36–1.52)	9
**Gray et al. 2018 [12]**	USA	Prospective population-based cohort study	3484	41	74	Dementia:1.13 (95%CI 0.82–1.56) AD: 1.11 (95% CI 0.77–1.61)	8
**Haenisch et al. 2015 [6]**	Germany	Longitudinal, multicenter cohort study	3076	34.9	79.7	Dementia:1.38 (95% CI 1.04–1.83) AD: 1.44 (95% CI 1.01–2.06)	9
**Hwang et al. 2018 [13]**	Korea	Population-based longitudinal study	70033	56.4	NA	Dementia: 0.99 (95% CI 0.70–1.39)	9
**Imfeld et al. 2018 [14]**	UK	Case-control study	82058	34.2	NA	Vascular Dementia: 1.22 (95% CI 1.05–1.42) AD: 0.88 (95% CI 0.80–0.97)	9
**Tai et al. 2017 [15]**	Taiwan	Case-control study	15726	59.1	55.5	Dementia: 1.22 (95% CI 1.05–1.42)	9
**Taipale et al. 2017 [16]**	Finland	nested case-control study	353576	34.8	80.8	AD: 1.03 (95% CI 1.00–1.05)	9

NA = not available, AD = Alzheimer’s disease, NOS = Newcastle–Ottawa Quality Assessment Scale

### Association of PPIs and risk of dementia and AD

**Fig 2:**
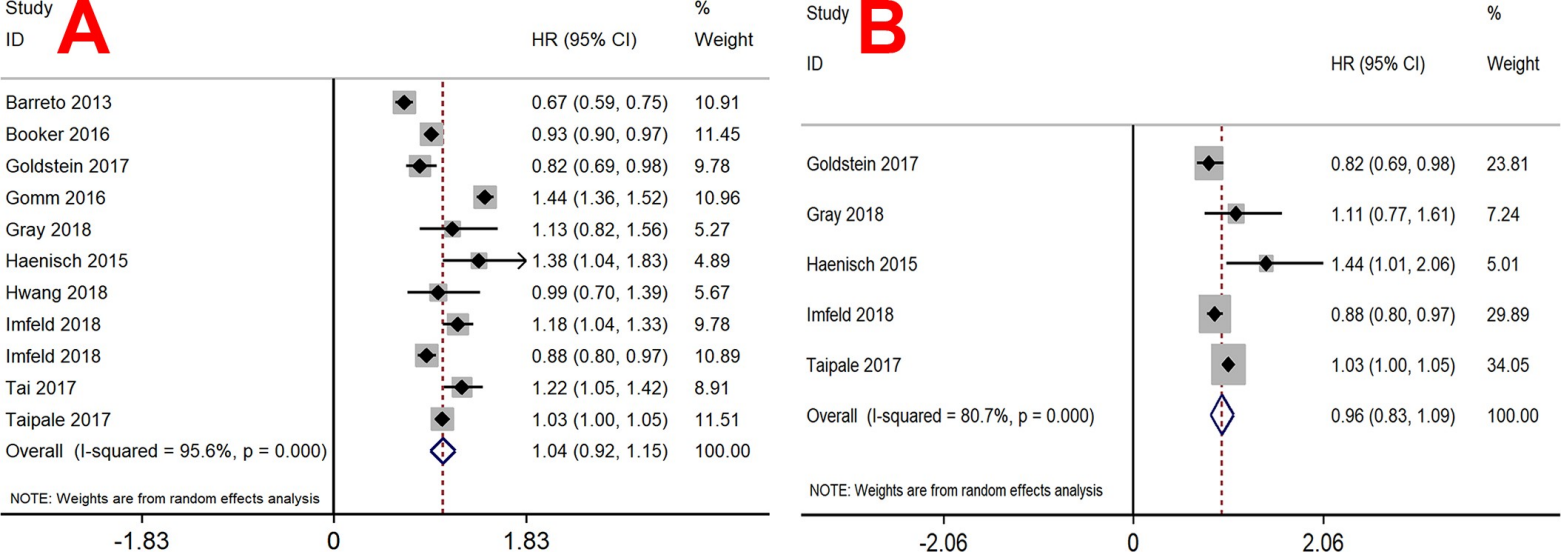
Forest plot of the association of PPIs and risk of dementia and AD. (A) dementia; (B) AD.

Ten studies that involved 642305 participants assessed the association of PPIs and risk of dementia, and PPI users were unassociated with all-cause dementia (HR = 1.04, 95% CI 0.92–1.15; *I*^2^ = 95.6%; *p* < 0.001; Fig 2A) in a random-effect model. Five studies that involved 421079 participants assessed the association of PPIs and risk of AD, and PPIs users were unassociated with AD (HR = 0.96, 95% CI 0.83–1.09; *I*^2^ = 80.7%; *p* <0 .001; Fig 2B) in a random-effect model.

### Heterogeneity and metaregression analysis

The heterogeneity among included studies was addressed. For dementia, we performed metaregression analyses by sample size (≤53525 or >53525), ethnicity (Europe or Non-Europe) and publication year (before 2016 or after 2016). For AD, we performed metaregression analyses by sample size (≤84216 or >84216), ethnicity (Europe or Non-Europe) and publication year (before 2016 or after 2016). Nonetheless, the source of heterogeneity remained unclear in the metaregression (Table 2).

**Table 2:** Metaregression analysis of potential sources of heterogeneity.

	Heterogeneity factors	Coefficient	SE	Z	P-value	95% CI (lower limit, upper limit)
**Dementia**						
	Sample size	0.159	0.182	0.88	0.410	−0.271, 0.589
	Publication year	−0.031	0.198	−0.16	0.880	−0.498,0.437
	Ethnicity	0.026	0.205	0.13	0.902	−0.459, 0.512
**AD**						
	Sample size	−0.119	0.513	−0.23	0.854	−6.642, 6.403
	Publication year	−0.010	0.398	−0.25	0.843	−5.152, 4.052
	Ethnicity	−0.163	0.398	0.41	0.752	−5.216, 4.889

AD = Alzheimer’s Disease, CI = confidence interval

Subgroup analyses was additionally performed (Table 3). For dementia, we performed subgroup analyses by study design (Cohort study or Non-cohort study) and male percentage (≤36.9 or >36.9). For AD, a subgroup analyses was performed by study design (Cohort study or Non-cohort study) and male percentage (≤34.8 or >34.8). However, we were unable to determine the possible heterogeneity causes via the abovementioned subgroup analyses.

**Table 3:** Subgroup analysis.

		HR (95% CI)	*P*	*I*^2^ (*P* value)
**Dementia**				
	**Study design**			
	Cohort study	1.15 (0.81–1.49)	<0.001	93.1% (<0.001)
	Non-cohort study	0.97 (0.86–1.07)	<0.001	94.8% (<0.001)
	**Male percentage (%)**			
	>36.9	0.99 (0.86–1.12)	<0.001	68.7% (0.013)
	≤36.9	1.08 (0.87–1.28)	<0.001	97.4% (0.059)
**AD**				
	**Study design**			
	Cohort study	1.06 (0.70–1.41)	<0.001	67.6% (0.067)
	Non-cohort study	0.96 (0.81–1.11)	<0.001	90.9% (0.010)
	**Male percentage**			
	>34.8	1.06 (0.70–1.41)	<0.001	67.6% (0.067)
	≤34.8	0.96 (0.81–1.11)	<0.001	90.9% (0.010)

AD = Alzheimer’s Disease, CI = confidence interval

### Sensitivity analysis and publication bias

**Fig 3:**
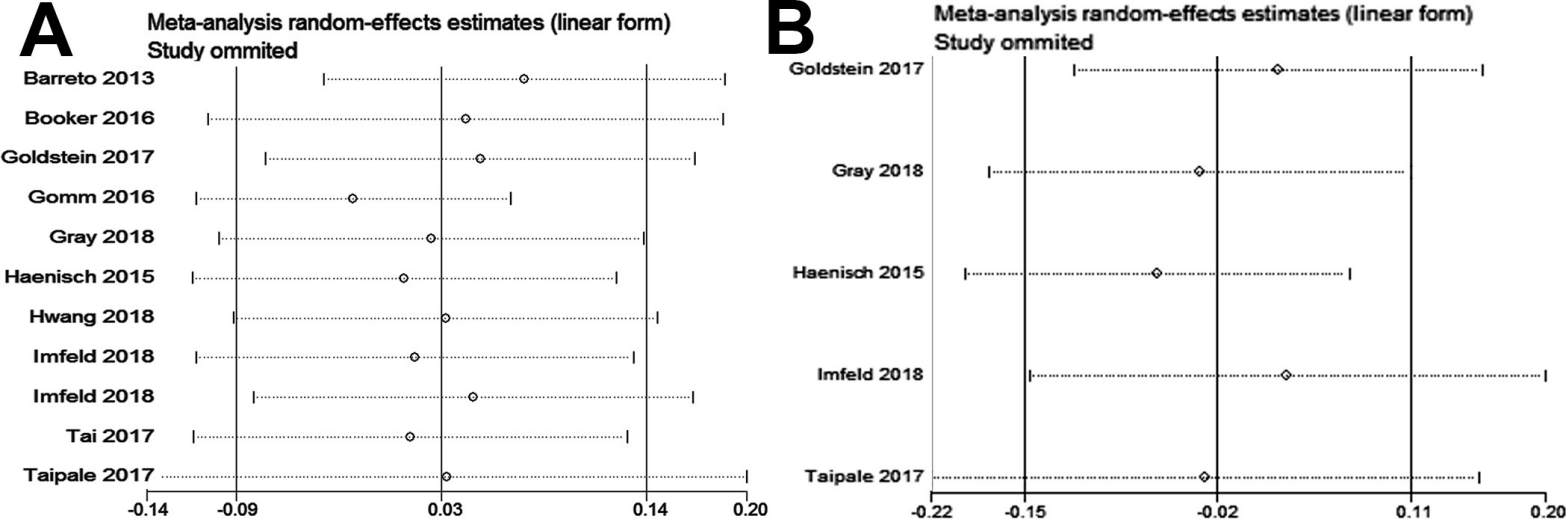
Funnel plot of sensitivity analysis. (A) dementia; (B) AD.

**Fig 4:**
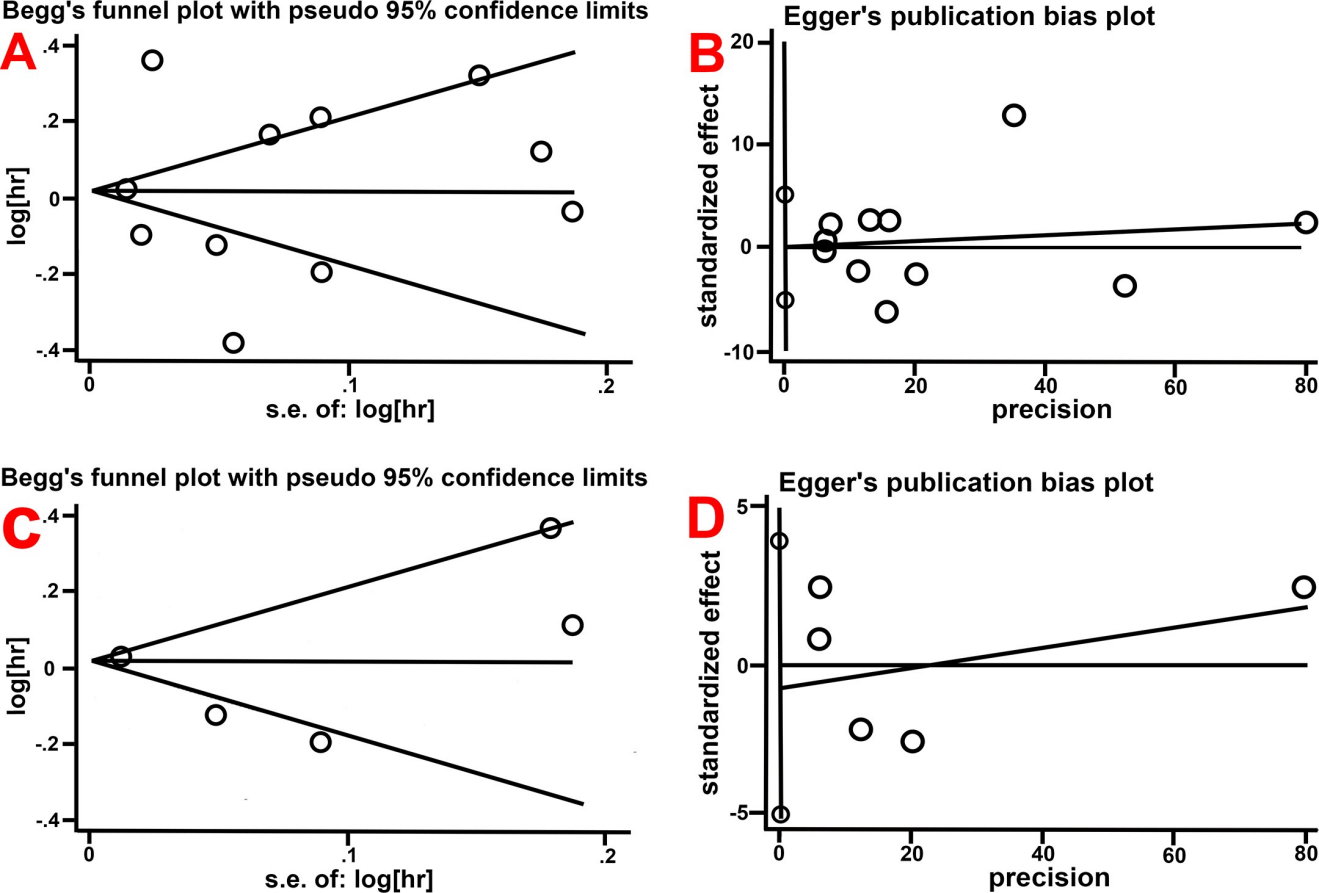
Funnel plot of publication bias. (4A) Begg’s test for all-cause dementia; (4B) Egger’s test for all-cause dementia, (4C) Begg’s test for AD; (4D) Egger’s test for AD.

Sensitivity analyses were performed to identify the influence of each individual study on the pooled results. The sensitivity analysis results suggested that our meta-analysis was stable (Dementia Fig 3A; AD, Fig 3B). No evidence of publication bias was detected for all-cause dementia (Begg’s test, p = 1.000, Fig 4A; Egger’s test, p = 0.982, Fig 4B) and for AD (Begg’s test, p = 1.000, Fig 4C; Egger’s test, p = 0.647, Fig 4D).

## Discussion

Previous studies that have assessed the associations of the use of PPIs and the risk of dementia and AD have reported inconsistent conclusions which cause constant worry on PPI use for augmentation strategy in clinical practice. Given the enormous public health implications of widespread PPI use, a thorough understanding of potential cognitive risks of cumulative PPI use is urgently needed. Thus, we performed a meta-analysis and provided the most up-to-date epidemiological evidence supporting that PPI use is unassociated with dementia and AD.

Several potential mechanisms have been proposed for the associations between PPI use and cognitive harm. PPI use results in vitamin B12 deficiency, which has been associated with cognitive dysfunction [23]. PPI use might also deleteriously affect cognition by enhancing deposition of β-amyloid in the brain, which is a pathologic hallmark of AD [24]. The theory behind the supposed connections seems plausible. Development of dementia via amyloid plaque production is likely to take many years; thus, we believe asserting that PPI avoidance may prevent dementia with a long follow-up period is appropriate [25]. Compared with nonusers, PPI users may be more susceptible to adverse events because they potentially have more comorbidities and are likely to be taking more medications than nonusers do [25, 26]; failing to account for high-risk medication classes, including benzodiazepines, narcotics and anticholinergic agents, associated with memory prohibits attributing the observed risk to PPIs alone.

We attempted to perform a metaregression analysis by ethnicity, publication year and sample size, but this analysis cannot explain the heterogeneity observed among the studies. In addition, the possible causes of heterogeneity were not determined through subgroup analyses by study design and male percentage. Heterogeneity may be attributed to the potential confounding due to covariate differences, variations in the life-style habits, and environmental factors; however, these were not analyzed in the present study due to the insufficiency of related data. Sensitivity analysis proved that the results of this meta-analysis were statistically reliable. Therefore, a methodologically preferable design, such as using population-based controls, is crucial to avoid selection bias and heterogeneity.

The first research highlight of this meta-analysis is its large sample size. The large number of total cases provided high statistical power to quantitatively evaluate the association between dementia and AD. Nevertheless, the present work has limitations that need to be considered. Firstly, only published studies were included. The exclusion of unpublished papers, abstracts and letters to the editor may lead to potential publication and reporting bias because positive results are likely to be accepted by journals. Secondly, we only selected literature that was written in English, which may have resulted in a language or cultural bias. Thirdly, most of the eligible studies were case-control studies, which were less conclusive than cohort studies and might introduce selection and recall biases.

In conclusion, our current evidence indicates that PPI use does not increase dementia and AD risk. More clinical studies with well-designed are needed to evaluate and establish the direct cause and effect relationships between PPI use and incident dementia. We recommend that physicians should regularly reconsider the appropriateness of treatment if long-term treatment is required.

## Supporting information

S1 Table PRISMA checklist. (DOCX)

S2 Table PRISMA flow diagram. (DOCX)
