# Supplementary material for: Proton pump inhibitor use does not increase dementia and Alzheimer’s disease risk: An updated meta-analysis of published studies involving 642305 patients
Source: PLoS One. 2019 Jul 2;14(7):e0219213. doi: 10.1371/journal.pone.0219213 (PMC6605652; doi:10.1371/journal.pone.0219213)
Supplement: S2 Table — (DOCX) [file pone.0219213.s002.docx]

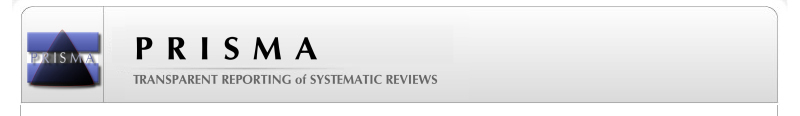
**PRISMA 2009 Flow Diagram**

**Records after** **duplicates removed
(n = 1941)**

**Full-text articles excluded,**

**insufficient data (n = 2)**

**duplicates (n = 1)**

**Records screened
(n = 817)**

**Studies included in quantitative synthesis (meta-analysis) (n = 10 )**

**Full-text articles assessed for eligibility
(n = 13)**

**Studies included in qualitative synthesis (n = 10)**

**Records excluded
(n = 814)**

## Included

## Eligibility

## Screening

## Identification

**Additional records identified through other sources (n = 857)**

**Records identified through database searching (n = 1901 )**
